# ERAD of proteins containing aberrant transmembrane domains requires ubiquitylation of cytoplasmic lysine residues

**DOI:** 10.1242/jcs.171215

**Published:** 2015-11-15

**Authors:** Kit Briant, Yee-Hui Koay, Yuka Otsuka, Eileithyia Swanton

**Affiliations:** Faculty of Life Sciences, University of Manchester, Oxford Road, Manchester M13 9PT, UK

**Keywords:** ER-associated degradation, ER quality control, Membrane protein, Retrotranslocation, Transmembrane domains, Ubiquitin

## Abstract

Clearance of misfolded proteins from the endoplasmic reticulum (ER) is mediated by the ubiquitin-proteasome system in a process known as ER-associated degradation (ERAD). The mechanisms through which proteins containing aberrant transmembrane domains are degraded by ERAD are poorly understood. To address this question, we generated model ERAD substrates based on CD8 with either a non-native transmembrane domain but a folded ER luminal domain (CD8^TMD*^), or the native transmembrane domain but a misfolded luminal domain (CD8^LUM*^). Although both chimeras were degraded by ERAD, we found that the location of the folding defect determined the initial site of ubiquitylation. Ubiquitylation of cytoplasmic lysine residues was required for the extraction of CD8^TMD*^ from the ER membrane during ERAD, whereas CD8^LUM*^ continued to be degraded in the absence of cytoplasmic lysine residues. Cytoplasmic lysine residues were also required for degradation of an additional ERAD substrate containing an unassembled transmembrane domain and when a non-native transmembrane domain was introduced into CD8^LUM*^. Our results suggest that proteins with defective transmembrane domains are removed from the ER through a specific ERAD mechanism that depends upon ubiquitylation of cytoplasmic lysine residues.

## Abstract (precis)

**Summary:** Proteins containing defective transmembrane domains are removed from the endoplasmic reticulum through a specific mechanism that depends upon the ubiquitylation of cytoplasmic lysine residues.

## INTRODUCTION

Integral membrane proteins comprise up to one-third of the human proteome (von Heijne and Gavel, 1988) and their biosynthesis involves a complex series of events including the integration of transmembrane domains (TMDs) into the lipid bilayer, folding of domains on both sides of the endoplasmic reticulum (ER) membrane, and, for multispanning or oligomeric proteins, the assembly of TMDs within the bilayer (Christis et al., 2008; Fiedler et al., 2010). Membrane proteins therefore represent a particular challenge to ER folding and quality control systems, and, perhaps unsurprisingly, many human diseases are linked to the misfolding and/or misassembly of membrane proteins (Ng et al., 2012). Misfolded proteins can disrupt ER function, and therefore it is essential that those which fail to fold or assemble correctly are quickly and efficiently removed from the ER. This is predominantly achieved through a process known as ER-associated degradation (ERAD), whereby the protein is moved back across the ER membrane for degradation by the 26S proteasome in the cytoplasm (Christianson and Ye, 2014; Vembar and Brodsky, 2008).

ERAD is initiated by recognition of the terminally misfolded protein (ERAD substrate), followed by movement across the ER membrane (retrotranslocation), ubiquitylation, extraction of the substrate from the ER membrane (dislocation) and finally targeting to the proteasome for degradation (Christianson and Ye, 2014; Ruggiano et al., 2014). These processes are mediated by a variety of ER and cytoplasmic factors that are organised around membrane-embedded E3 ubiquitin ligase complexes, which catalyse polyubiquitylation of the substrate protein and facilitate movement of the polypeptide across the ER membrane (Christianson and Ye, 2014; Ruggiano et al., 2014). Distinct combinations of ERAD factors are required for degradation of different misfolded proteins, and a key unresolved question is what dictates the requirements for degradation of the huge variety of potential ERAD substrates generated by misfolding of diverse membrane proteins. In *Saccharomyces cerevisiae*, the location of the folding defect is a key determinant, with distinct pathways mediating degradation of proteins with misfolded lesions in the cytoplasm (ERAD-C), membrane (ERAD-M) and lumen (ERAD-L) (Ruggiano et al., 2014). Although analogous pathways might exist in mammals, the increased number of ERAD factors, E3 ligases and potential substrates has made attempts to extrapolate these findings to mammals difficult (Christianson and Ye, 2014). Thus, defining the mechanisms through which ERAD substrates with different topologies and structural defects are recognised and retrotranslocated remains a key goal in the field.

Similar to the ERAD-L pathway described in yeast, luminal regions of membrane proteins are scrutinised by molecular chaperones such as BiP (also known as HSPA5) and lectins, including OS-9, which use exposed hydrophobic sequences and glycan-based signals, respectively, to identify misfolded conformations (Alcock and Swanton, 2009; Bernasconi et al., 2010; Burr et al., 2013; Christianson et al., 2008; Geiger et al., 2011; Otero et al., 2010) and hand them over to ERAD E3 ligase complexes that mediate retrotranslocation and polyubiquitylation. By definition, folding defects within the lipid bilayer cannot be recognised by luminal factors, and very little is known about how proteins containing non-native TMDs (potential ERAD-M substrates) are identified, ubiquitylated and removed from the ER of mammalian cells. These questions have been difficult to address in the absence of well-defined model ERAD-M substrates such as those that have allowed characterisation of the luminal quality control and ERAD machinery. The canonical mammalian ERAD-M substrate, TCRα, a type I transmembrane glycoprotein previously thought to contain a TMD-based signal for ER retention and degradation, was recently shown to translocate entirely into the ER lumen, leading to recognition by BiP and degradation through an ERAD-L type pathway (Feige and Hendershot, 2013; Shin et al., 1993). Thus, additional models are needed in order to address how the folding and assembly status of TMDs within the lipid bilayer is monitored, and define the mechanisms that mediate recognition and retrotranslocation of proteins containing non-native TMDs. To this end, we have developed a chimeric model ERAD-M substrate by inserting an exogenous TMD into the type I membrane protein CD8α. We show that this chimera has a folded extracellular and luminal domain, and that the non-native TMD causes retention by ER quality control (ERQC) and degradation through ERAD. In contrast to CD8α possessing a misfolded luminal domain, dislocation and degradation of the TMD chimera required ubiquitylation of lysine residues located in the cytoplasmic tail. Degradation of a second ERAD substrate also containing an unassembled TMD was similarly dependent upon cytoplasmic lysine residues. Our results indicate that proteins containing defective TMDs are removed from the ER through a specific ERAD pathway that is mechanistically distinct from that which mediates degradation of membrane proteins with luminal folding defects.

## RESULTS

### Design and characterisation of a model protein to study transmembrane domain quality control

**Fig. 1.**
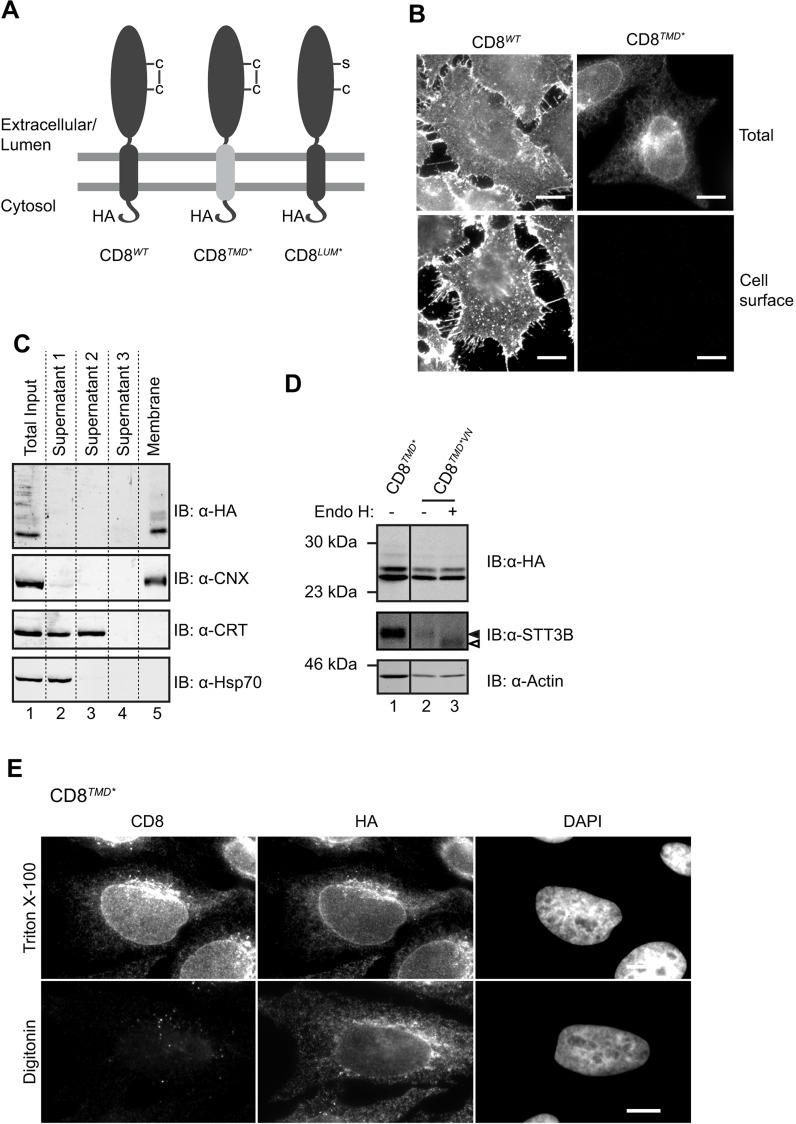
CD8^TMD*^ is integrated into the membrane with the correct topology. (A) Cartoon of CD8 chimeras used. (B) Stable HeLa cell lines expressing CD8*^WT^* or CD8^TMD*^ were fixed and labelled for total (permeabilised cells, anti-HA antibodies) and cell surface (intact cells, anti-CD8 antibodies) CD8. Images were collected in parallel using equal exposure times. (C) Cells expressing CD8^TMD*^ were lysed and subjected to carbonate extraction. Equivalent amounts of supernatant 1 (initial lysate supernatant), 2 and 3 (from sequential carbonate extractions) and the final membrane pellet were analysed by immunoblotting (IB) with antibodies against HA, calnexin (CNX), calrecticulin (CRT) and Hsp70. (D) Lysates of cells expressing CD8^TMD*^ or CD8^TMD*VN^ were treated with or without EndoH and analysed by immunoblotting with antibodies against HA, STT3B and actin. Closed arrowhead indicates N-glycosylated protein, open arrowhead indicates deglycosylated protein. (E) Cells expressing CD8^TMD*^ were permeabilised with digitonin or Triton-X 100 and labelled with antibodies against the extracellular (luminal) domain of CD8 and the cytoplasmic HA epitope. DNA was stained with DAPI. Scale bars: 10 µm.

In order to study the quality control mechanisms that specifically monitor TMDs in mammalian cells, we first needed to develop a suitable model protein that has defective transmembrane segment(s) but properly folded cytoplasmic and luminal domains. To this end, we replaced the endogenous TMD of the type I membrane protein CD8α (hereafter CD8) with one of the TMDs from a multi-pass membrane protein, proteolipid protein (PLP, also known as PLP1) and generated tetracycline-inducible stable cell lines expressing HA-tagged CD8 variants (Fig. 1A). We reasoned that a single TMD from a multi-spanning protein, such as PLP, would expose non-native features such as polar residues that would normally be masked upon folding of full-length PLP (Ng et al., 2012; Swanton et al., 2003), and would therefore mimic a misassembled TMD when inserted into CD8. Indeed, whereas wild-type CD8-HA (CD8*^WT^*) was transported to the plasma membrane of HeLa cells as expected for the correctly folded protein (Fig. 1B, left), CD8 containing the fourth TMD from PLP (CD8^TMD*^) failed to reach the cell surface (Fig. 1B, bottom right). Instead, the chimeric protein was retained intracellularly (Fig. 1B, top right), suggesting that the presence of the non-native TMD caused recognition by the cellular quality control machinery.

The TMD sequence inserted into CD8^TMD*^ contains several polar residues (see Materials and Methods) and, because it has recently been shown that less-hydrophobic TMDs can completely enter the ER lumen (Feige and Hendershot, 2013; Shin et al., 1993), we examined whether this was the case for CD8^TMD*^. However, CD8^TMD*^ was present exclusively in the membrane fraction following carbonate extraction together with the ER-resident membrane protein calnexin (Fig. 1C, lane 5), suggesting that it was stably integrated into the membrane. Cytoplasmic Hsp70s and the ER luminal protein calreticulin were recovered in the supernatant (Fig. 1C, lanes 2 and 3) but not the membrane fractions (Fig. 1C, lane 5), demonstrating the efficiency of the carbonate extraction. To further confirm that CD8^TMD*^ was not fully translocated into the ER lumen, an artificial N-glycosylation consensus site was introduced into the C-terminal cytoplasmic domain of CD8^TMD*^ by replacement of a valine at position 222 for an asparagine residue (CD8^TMD*VN^). Complete translocation into the ER lumen would expose this site to the oligosaccharyltransferase complex, allowing glycosylation and increasing its mobility on SDS-PAGE (cf. Feige and Hendershot, 2013). However, the migration of CD8^TMD*VN^ was not altered relative to CD8^TMD*^ (Fig. 1D, compare lanes 1 and 2), and was also unchanged following treatment with endoglycosidase H to remove high-mannose N-glycans (Fig. 1D, lanes 2 and 3; note the clear shift in the mobility of the endogenous glycoprotein STT3B demonstrating the effectiveness of this treatment). Thus, the C-terminal domain of CD8^TMD*^ does not appear to enter the ER lumen. The accessibility of the cytoplasmic HA epitope upon selective permeabilisation provided further evidence that CD8^TMD*^ was correctly oriented in the membrane (Fig. 1E), given that it could be detected with anti-HA antibodies in digtonin-permeabilised cells in which the ER and secretory organelles remains intact (Fig. 1E, bottom; Fig. S1A). In contrast, staining with an anti-CD8 antibody, which recognises an epitope in the extracellular (luminal) domain of CD8 was only apparent when intracellular membranes were permeabilised with Triton X-100 (Fig. 1E, top). Therefore, we conclude that CD8^TMD*^ is integrated into the membrane, has the correct orientation (HA located in the cytoplasm and the CD8 within the lumen; Fig. 1A), and that the presence of the engineered TMD causes it to be retained intracellularly.

In order to confirm that the extracellular domain of CD8^TMD*^ was not misfolded, we examined whether CD8^TMD*^ was recognised by BiP, an Hsp70 chaperone known to bind exposed hydrophobic patches on unfolded proteins within the ER lumen (Blond-Elguindi et al., 1993; Flynn et al., 1991). To provide a control, we generated a version of CD8 (Fig. 1A, CD8^LUM*^) in which folding of the extracellular domain was disrupted by mutagenising a cysteine residue known to form an intramolecular disulphide bond in the extracellular domain (Leahy et al., 1992). Co-immunoprecipitation revealed nearly twice as much BiP bound to CD8^LUM*^ compared to CD8^TMD*^ (Fig. S1B,C), suggesting that CD8^TMD*^ exposed far fewer BiP-binding sites than CD8^LUM*^. Native CD8α forms homodimers through interchain disulphide bonds (Leahy et al., 1992), and these were apparent in cells expressing CD8*^WT^* as a 55-kDa species that was sensitive to reducing agents (Fig. S1D). CD8^TMD*^ also migrated as a higher molecular mass form of ∼45 kDa that was lost upon reduction with dithiothreitol (Fig. S1D), consistent with formation of disulphide-linked dimers. The monomeric forms of both CD8*^WT^* and CD8^TMD*^ migrated more rapidly under non-reducing conditions (Fig. S1D), suggesting that the extracellular domain of both proteins underwent oxidative folding. Thus, CD8^TMD*^ appears to undergo conformational maturation up to and including formation of inter- and intra-molecular disulphide bonds, indicating that the presence of the non-native TMD does not cause misfolding of the extracellular domain. Neither CD8^LUM*^ nor a variant possessing a second luminal mutation (CD8*^G111S^*) could be detected by an anti-CD8 monoclonal that efficiently labelled CD8*^WT^* (Fig. S1E,F). In contrast, robust staining of CD8^TMD*^ was observed (Fig. S1E,F), providing further evidence that the extracellular domain of the chimera was properly folded. However, given that the epitope for this antibody is not known, a caveat to this interpretation is that the mutated cysteine residue in CD8^LUM*^ could form part of this epitope.

Taken together, these results show that CD8^TMD*^ is integrated into the membrane, correctly oriented, and possesses a folded extracellular domain. Thus, we conclude that determinants located in the non-native TMD sequence are responsible for intracellular retention of CD8^TMD*^. Therefore, CD8^TMD*^ represents a suitable model protein with which to study the quality control of TMDs in the secretory pathway.

### CD8^TMD*^ is localised to the ER and degraded through ERAD

**Fig. 2.**
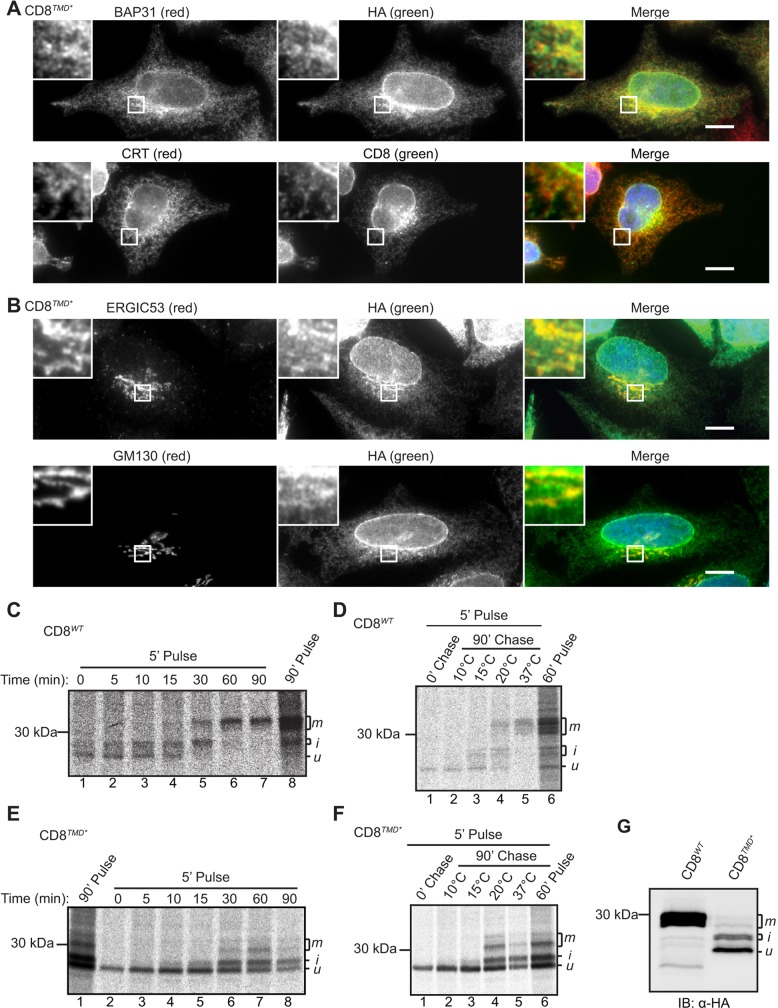
CD8^TMD*^ is localised to the ER at steady state but partially escapes to the Golgi. (A) Cells expressing CD8^TMD*^ were fixed and labelled with anti-HA and anti-BAP31, or mouse anti-HA and anti-calreticulin antibodies. (B) Cells expressing CD8^TMD*^ were fixed and labelled with anti-HA and anti-ERGIC53, or anti-HA and anti-GM130 antibodies. Scale bars: 10 µm. (C,E) Cells expressing CD8*^WT^* or CD8^TMD*^ were pulse-labelled with [^35^S]Met/Cys for 5 min or 90 min as indicated and chased for up to 90 min in the presence of unlabelled Met and Cys. CD8 was immunoprecipitated with anti-HA antibodies, and analysed by phosphorimaging. (D,F) Cells expressing CD8*^WT^* or CD8^TMD*^ were pulse-labelled with [^35^S]Met/Cys for or 5 or 60 min and chased for 90 min at the indicated temperature and analysed as above. (G) Lysates of cells expressing CD8*^WT^* or CD8^TMD*^ were analysed by immunoblotting with anti-HA antibodies. u, i and m indicate the unglycosylated precursor, an initially glycosylated intermediate, and the mature glycoform of CD8, respectively.

At steady state, CD8^TMD*^ exhibited a reticular distribution typical of the ER and showed a high degree of colocalisation with the ER marker proteins BAP31 (also known as BCAP31) and calreticulin (Fig. 2A), suggesting that the non-native TMD was recognised and retained by ERQC systems. In addition, a proportion of CD8^TMD*^ colocalised with markers of the ER-Golgi intermediate compartment (ERGIC) and Golgi complex (Fig. 2B), indicating that some CD8^TMD*^ might be able exit the ER and reach later stages in the secretory pathway. Following exit from the ER, CD8 undergoes O-glycosylation in the Golgi (Gill et al., 2011; Jackson et al., 1993; Pascale et al., 1992a,b), allowing the intracellular transport of CD8^TMD*^ to be followed by pulse-chase labelling. CD8*^WT^* was initially synthesised as a precursor of ∼25 kDa (Fig. 2C, lane 1) that was converted into higher molecular mass forms of ∼27–28 kDa, which were in turn replaced by a broad band at ∼30 kDa during the 90-min chase (Fig. 2C, lanes 2–7). These different forms have previously been identified as the unglycosylated precursor (u), an initially glycosylated intermediate (i) and the mature glycoform (m) of CD8, respectively (Pascale et al., 1992a,b). Consistent with this interpretation, most CD8*^WT^* remained in the ‘u’ form when the chase was carried out at either 10°C, to inhibit ER exit (Tartakoff, 1986), or 15°C, to inhibit transport beyond the ERGIC (Fig. 2D, lanes 2 and 3), whereas the ‘i’ and ‘m’ forms predominated after chasing at 20°C, which allows transport as far as the trans-Golgi network (Matlin and Simons, 1983), or at 37°C (Fig. 2D, lanes 4 and 5). CD8^TMD*^ was also observed as a single species immediately after the pulse (Fig. 2E, lane 2), and several additional higher molecular mass forms appeared after 15–30 min of chase, coinciding with a decrease in the intensity of the precursor (Fig. 2E, lanes 5–8). Their relative migration on SDS-PAGE and comparison with CD8*^WT^* suggested that these species represent the unmodified precursor and O-glycosylated ‘i’ and ‘m’ forms, respectively (Fig. 2E). Performing the chase at reduced temperature confirmed that the higher molecular mass forms were only produced under conditions that permit trafficking to the Golgi (Fig. 2F). At steady state, CD8^TMD*^ was observed primarily as the unmodified precursor with a smaller amount of the intermediate ‘i’ and very little, if any, of the ‘m’ form (Fig. 2G). Treatment of cells with brefeldin A to redistribute Golgi-resident enzymes to the ER, converted all the CD8^TMD*^ into higher molecular mass forms (Fig. S1G), providing further evidence that these represent O-glycosylated species. Taken together, these results show that CD8^TMD*^ is not statically retained in the ER because a proportion undergoes post-translational modification in the Golgi. This might be comparable to other non-native proteins that are known to partially escape the ER and subsequently undergo retrieval from the Golgi complex (Caldwell et al., 2001; Hammond and Helenius, 1994; Pan et al., 2011; Vashist et al., 2001).

**Fig. 3.**
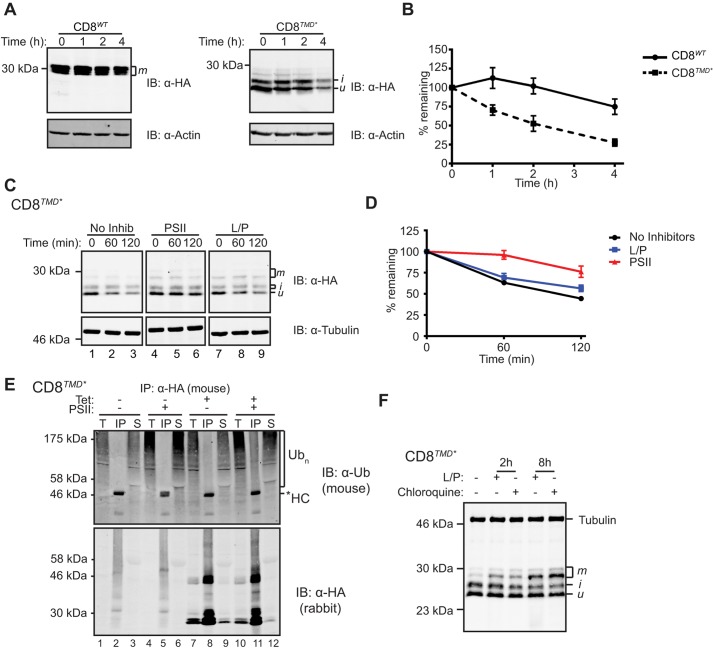
CD8^TMD*^ is an ERAD substrate. (A) Measurement of protein degradation by cycloheximide chase assays. Cells expressing CD8*^WT^* or CD8^TMD*^ were chased by incubation with cycloheximide for up to 4 h. Cell lysates were analysed by immunoblotting (IB) with anti-HA and anti-actin antibodies, followed by secondary antibodies labelled with infrared fluorophores. (B) The anti-HA antibody signal normalised relative to the anti-actin signal is expressed as a percentage of that present at the start of the chase. Graphs represent the mean±s.e.m. of three independent experiments. (C) Cells expressing CD8^TMD*^ were left untreated or treated with leupeptin and pepstatin A (L/P) or PSII for 2 h, then chased with cycloheximide in the continued presence of inhibitors for up to 2 h, then analysed as in A. (D) Protein levels were quantified as in B. (E) Cells were induced with tetracycline (tet) to express CD8^TMD*^ or left uninduced, treated with or without PSII for 8 h, then lysed and the CD8 immunoprecipitated (IP) with anti-HA antibodies. Samples were analysed by immunoblotting with anti-ubiquitin and anti-HA antibodies. *HC, IgG heavy chain; Ub_n_, polyubiquitylated proteins; T, 5% of the total input; IP, immunoprecipitated sample; S, supernatant after immunoprecipitation. (F) Cells expressing CD8^TMD*^ were left untreated, or treated with leupeptin and pepstatin (L/P) or chloroquine for the indicated time. Cell lysates were analysed by immunoblotting with anti-HA and anti-α-tubulin antibodies. u, i and m indicate the unglycosylated precursor, an initially glycosylated intermediate, and the mature glycoform of CD8, respectively.

Having established that CD8^TMD*^ was recognised and retained by quality control mechanisms in the early secretory pathway, we next examined whether the non-native TMD caused degradation of CD8 using cycloheximide chase experiments. Cells expressing CD8*^WT^* or CD8^TMD*^ were treated with cycloheximide to block protein synthesis, chased in the presence of cycloheximide for 0–240 min, then the amount of protein remaining at each time point was determined by immunoblotting. As shown in Fig. 3A, the level of CD8*^WT^* remained relatively constant over the 4-h chase, consistent with this being a stable plasma membrane protein. In contrast, CD8^TMD*^ was rapidly lost following addition of cycloheximide (Fig. 3A), suggesting that the chimera was degraded over time. Quantification revealed that the half-life of CD8^TMD*^ was ∼120 min compared to well over 240 min for the wild type (Fig. 3B). Aside from an initial increase in levels of the ‘i’ form, the two major forms of CD8^TMD*^ decreased with comparable kinetics (Fig. 3A). Importantly, degradation of CD8^TMD*^ was not due to its prolonged residence in the ER, because CD8 possessing a dilysine ER retrieval motif (CD8KKxx; Jackson et al., 1993) was stable despite being localised at the ER (Fig. S2A). Thus, in addition to causing retention in the ER, the non-native TMD present in CD8^TMD*^ constitutes a signal for rapid degradation.

In order to identify the pathways that mediate degradation of CD8^TMD*^, cycloheximide chase assays were carried out in the presence of a proteasome inhibitor (Z-LLF-CHO; PSII) or a combination of leupeptin and pepstatin A to inhibit lysosomal proteolysis (Fig. 3C,D). Treatment with PSII substantially slowed the loss of CD8^TMD*^ during the chase, with ∼75% of the protein remaining after 120 min compared to just 45% in the absence of inhibitors (Fig. 3C,D). Similar results were obtained with the proteasome inhibitors bortezomib and MG132 (Fig. S2B,C), suggesting that proteasomes mediate degradation of CD8^TMD*^. In contrast, the lysosomal inhibitors did not obviously alter the rate at which CD8^TMD*^ was lost during the 2-h chase period (Fig. 3C,D). Hence, CD8^TMD*^ is degraded primarily through a proteasomal route, which, given its ER localisation, is most likely to be the ERAD pathway. Notably, proteasome inhibition stabilised the Golgi modified ‘i’ form as well as the major ‘u’ form of CD8^TMD*^ (Fig. 3C). This indicates that CD8^TMD*^, which reached the Golgi, might ultimately be degraded through ERAD, supporting the view that retrieval mechanisms return some of the escaped protein to the ER. Consistent with this interpretation, CD8^TMD*^ accumulated in the ER of proteasome inhibitor-treated cells (Fig. S2D), as would be predicted for an ERAD substrate. Under these conditions, a prominent juxtanuclear localisation of CD8^TMD*^ was observed (Fig. S2D). This might reflect accumulation in the ER quality control compartment (ERQC), a subdomain of the ER specialised for the recognition and degradation of misfolded membrane and secretory proteins (Kamhi-Nesher et al., 2001; Leitman et al., 2014).

ERAD typically involves polyubiquitylation of substrate proteins prior to proteasomal degradation. To test whether CD8^TMD*^ was ubiquitylated, cells expressing the chimera were treated with PSII to block proteasomal degradation of ubiquitylated proteins, and CD8^TMD*^ was isolated by immunoprecipitation. Immunoblotting immunoprecipitated material with anti-ubiquitin antibodies revealed a broad smear of high-molecular-mass bands near the top of the gel, characteristic of polyubiquitin-conjugated proteins (Fig. 3E, lane 11). These species were only observed in immunoprecipitates from cells induced to express CD8^TMD*^ (Fig. 3E, lane 5) and were far less abundant in the absence of proteasome inhibitor treatment (Fig. 3E, lane 8), despite equal loading of immunoprecipitated CD8^TMD*^ (Fig. 3E, bottom panel). Thus, we conclude that CD8^TMD*^ is polyubiquitylated en route to proteasomal degradation, consistent with it being a substrate for ERAD.

Although leupeptin and pepstatin A had no obvious effect on the stability or levels of the unmodified ‘u’ or intermediate ‘i’ forms of CD8^TMD*^ in the short-term (Fig. 3C,F), we noticed that treatment with these inhibitors, or an alternative inhibitor chloroquine, caused a gradual accumulation of the higher-molecular-mass ‘m’ forms over time (Fig. 3F). Furthermore, CD8^TMD*^ could be observed in lysosomes following treatment with leupeptin and pepstatin A (Fig. S2E), suggesting that a fraction of the CD8^TMD*^ that escapes the ER is ultimately targeted to lysosomes for degradation.

Taken together, these results provide evidence that CD8^TMD*^ is primarily degraded through the ERAD pathway, with lysosomal degradation serving as a backup pathway to eliminate CD8^TMD*^, which evades ER quality control (i.e. escapes retention, retrieval and ERAD). Hence, we conclude that CD8^TMD*^ represents an authentic mammalian ERAD-M substrate given that it possesses a folded luminal domain, and determinants in its non-native TMD cause ER retention and proteasomal degradation.

### Ubiquitylation of cytoplasmic lysine residues is required for dislocation and degradation of CD8^TMD*^

**Fig. 4.**
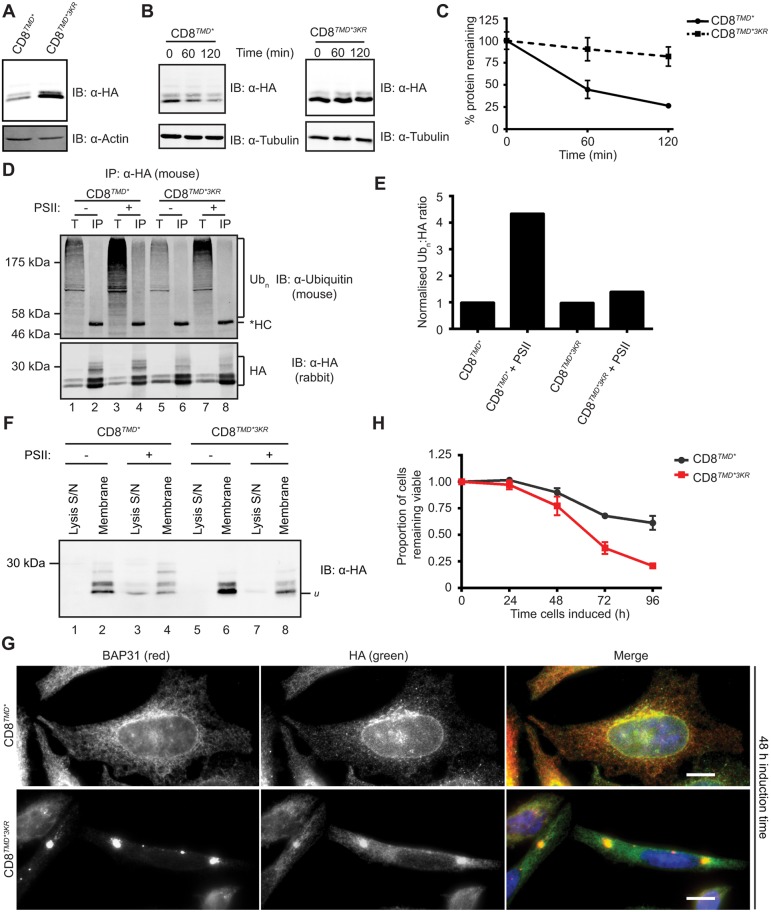
Ubiquitylation of cytoplasmic lysine residues is required for dislocation and degradation of CD8^TMD*^. (A) Lysates of cells expressing CD8^TMD*^ or CD8^TMD*3KR^ were analysed by immunoblotting (IB) with anti-HA and anti-actin antibodies. (B,C) Degradation of CD8^TMD*^ and CD8^TMD*3KR^ was measured by cycloheximide chase assays as in Fig. 3A,B. (D) Cells expressing CD8^TMD*^ or CD8^TMD*3KR^ were treated with or without PSII for 8 h, lysed and the CD8 immunoprecipitated (IP) with anti-HA antibodies. Samples were analysed by immunoblotting with anti-ubiquitin and anti-HA antibodies. *HC, IgG heavy chain; Ub_n_, polyubiquitylated proteins; T, 5% of the total input; IP, immunoprecipitated sample. (E) The anti-ubiquitin and anti-HA antibody signals from the immunoprecipitated samples were quantified and expressed as a ratio of Ub_n_:HA. (F) Cells expressing CD8^TMD*^ or CD8^TMD*3KR^ were left untreated or treated with PSII for 2 h prior to carbonate extraction as in Fig. 1C. Equivalent proportions of the initial lysis supernatant and the membrane fraction for each condition were analysed by immunoblotting with antibodies against HA. Loading controls and subcellular fractionation markers are shown in Fig. S3. u, the unglycosylated precursor of CD8. (G) Cells were induced to express CD8^TMD*^ or CD8^TMD*3KR^ for 48 h prior to fixation. Cells were labelled with anti-HA and anti-BAP31 antibodies. Scale bars: 10 µm. (H) Cells expressing CD8^TMD*^ or CD8^TMD*^^3KR^ were plated at the same time, induced at 24 h intervals up to a maximum total induction time of 96 h or left uninduced, and viable cells determined by MTT assays. The amount of viable cells remaining after induction was expressed relative to cultures that remained uninduced throughout the 96 h. Graphs represent the mean±s.e.m. of three independent experiments.

In order to characterise the requirements for degradation of CD8^TMD*^, we next examined the target sites for ubiquitylation. The cytoplasmic domain of CD8^TMD*^ contains three lysine residues, and replacement of these with arginine residues (generating CD8^TMD*3KR^) led to a striking increase in the steady-state expression levels of the chimera (Fig. 4A). Cycloheximide chase assays revealed that CD8^TMD*3KR^ was almost completely stable over the 2-h chase (Fig. 4B,C), suggesting that ubiquitylation of cytoplasmic lysine residues is required for ERAD of CD8^TMD*^. Indeed, very little polyubiquitylated CD8^TMD*3KR^ was detected, even after treatment of cells with proteasome inhibitor, when ubiquitylated CD8^TMD*^ was clearly observed (Fig. 4D, compare lanes 4 and 8; Fig. 4E). Taken together, these results provide evidence that ubiquitylation of CD8^TMD*^ on cytoplasmic lysine residues is a crucial step in the degradation of this ERAD-M substrate.

In addition to marking ERAD substrates for proteasomal degradation, ubiquitylation might be required for extraction of substrates from the ER membrane. We therefore examined whether cytoplasmic lysine residues were required for dislocation of CD8^TMD*^ into the cytoplasm. Cells expressing CD8^TMD*^ or CD8^TMD*3KR^ were treated with or without proteasome inhibitor, then subjected to subcellular fractionation and carbonate extraction in order to separate protein that had undergone dislocation from that which remained integrated in the ER membrane (see Fig. S3A,B for loading controls showing the effectiveness of this fractionation). In the absence of proteasome inhibition, neither CD8^TMD*^ nor CD8^TMD*3KR^ were observed in the supernatant fraction (Fig. 4F, lane 1 and 5). Treatment with PSII led to the appearance of CD8^TMD*^ in the supernatant fraction (Fig. 4F, lane 3), showing that the dislocated protein accumulated in the cytoplasm when proteasomal degradation was inhibited. In contrast, almost no CD8^TMD*3KR^ was found in the supernatant after proteasome inhibitor treatment (Fig. 4F, lane 7, note approximately equal loading of the chimeras, also shown in Fig. S3A,B), and the protein remained predominantly in the membrane fraction (Fig. 4F, lane 8), suggesting that the majority of the KR mutant had failed to undergo dislocation. Hence, we conclude that ubiquitylation of cytoplasmic lysine residues is an early event in CD8^TMD*^ degradation and is required for dislocation into the cytoplasm.

The observation that mutation of the cytoplasmic lysine residues effectively inhibited CD8^TMD*^ degradation allowed us to examine the consequences of failing to remove protein containing aberrant TMDs from the ER. Like CD8^TMD*^, CD8^TMD*3KR^ was distributed throughout the ER after 24 h of expression, as shown by colocalisation with BAP31 (Fig. S3E). The subcellular distribution of CD8^TMD*^ did not change dramatically over time following induction with tetracycline, and remained dispersed through the ER (Fig. 4G, top). In contrast, CD8^TMD*3KR^ had a strikingly different localisation after 48 h of continued expression, appearing in large intracellular inclusions (Fig. 4G, bottom). These structures were positive for the ER marker BAP31, consistent with CD8^TMD*3KR^ failing to undergo dislocation into the cytoplasm and thus remaining in the ER membrane. Although the precise nature of these inclusions is not known, they might represent a subcompartment of the ER containing aggregates of CD8^TMD*3KR^ (Fu and Sztul, 2003; Kamhi-Nesher et al., 2001; Leitman et al., 2014; Tanaka et al., 2002; Valetti et al., 1991). Cells containing CD8^TMD*3KR^ puncta showed clear changes in overall morphology, appearing much smaller and thinner, suggesting puncta formation is associated with disruption of cellular homeostasis. Therefore, we examined the effect of expressing CD8^TMD*3KR^ on cell viability. Cells were grown for 96 h, and induced to express CD8^TMD*^ or CD8^TMD*3KR^ at different time points or left uninduced. After a total of 96 h, the number of viable cells was determined using MTT assays. Expression of CD8^TMD*^ was somewhat detrimental to cell growth, with the number of viable cells being ∼70% of that in uninduced cultures after 96 h (Fig. 4H). CD8^TMD*3KR^, however, had a very pronounced effect on cell viability. Cultures expressing CD8^TMD*3KR^ for 96 h had only∼25% of the viable cell number of uninduced cultures (Fig. 4H), suggesting that accumulation of this ERAD resistant chimera inhibited cell growth and/or induced cell death. These results highlight the importance of the ERAD-M pathway for removing proteins containing aberrant TMDs, such as CD8^TMD*^, and maintaining cellular homeostasis.

### Distinct mechanisms mediate degradation of ERAD substrates containing defective TMDs

**Fig. 5.**
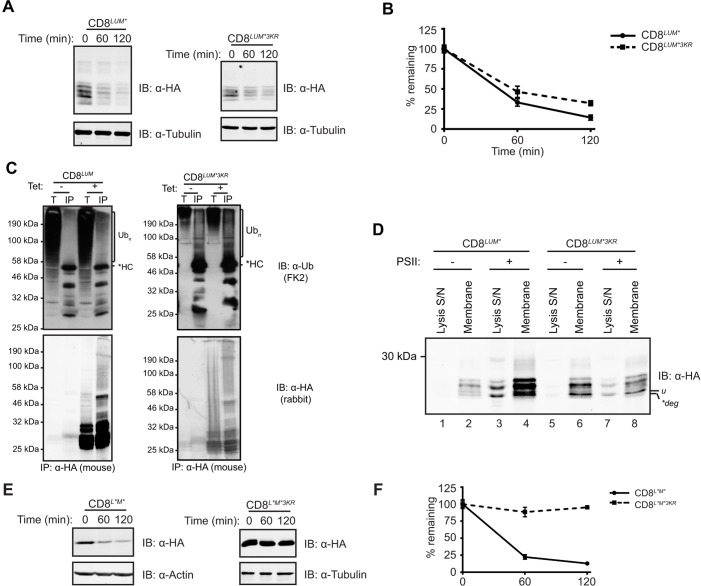
**Cytoplasmic lysine residues are not required for degradation of CD8 containing only a luminal folding defect.** (A,B) Degradation of CD8^LUM*^ and CD8^LUM*3KR^ was examined by cycloheximide chase assays as in Fig. 3A,B. (C) Cells were induced with tetracycline (tet) or left uninduced, then lysed and the CD8 immunoprecipitated (IP) with anti-HA antibodies. Samples were analysed by immunoblotting with anti-ubiquitin and anti-HA antibodies. *HC, IgG heavy chain; Ub_n_, polyubiquitylated proteins; T, 5% of the total input; IP, immunoprecipitated sample. (D) Dislocation of CD8^LUM*^ and CD8^LUM*3KR^ was examined as in Fig. 4F. u, the unglycosylated precursor of CD8; *deg, degradation product. (E,F) Degradation of CD8^L*M*^ and CD8^L*M*3KR^ was examined by cycloheximide chase assays as in Fig. 3A,B.

Next, we examined whether the location of the folding defect in different ERAD substrates determines the initial site of ubiquitylation. To address this, we exploited CD8^LUM*^, which possesses a misfolded luminal domain (Fig. 1A; Fig. S1B,E). Like CD8^TMD*^, CD8^LUM*^ was localised to the ER and Golgi (Fig. S4A), received Golgi modification to the ‘i’ form (Fig. S4B,C), became ubiquitylated (Fig. 5C) and was rapidly degraded, at least in part, through a proteasome-dependent pathway (Fig. 5A,B; Fig. S4E,F). However, in direct contrast to CD8^TMD*^, CD8^LUM*^ was not dramatically stabilised by mutation of the cytoplasmic lysine residues (Fig. 5A), and both CD8^LUM*^ and CD8^LUM*3KR^ were degraded with a comparable half-life of ∼60 min (Fig. 5B). Furthermore, neither ubiquitylation (Fig. 5C) nor dislocation (Fig. 5D, compare lanes 3 and 7; Fig. S3C,D) of CD8^LUM*^ were prevented by mutation of the cytoplasmic lysine residues. Thus, ubiquitylation of cytoplasmic lysine residues is not essential for dislocation and degradation of CD8^LUM*^, as demonstrated for major histocompatibility complex (MHC) class I molecules, which also contains a luminal degron (Burr et al., 2013).

Taken together, these results provide evidence that distinct mechanisms mediate degradation of CD8^TMD*^ and CD8^LUM*^, and suggest a model whereby the site of the misfolded region dictates the ERAD pathway followed by different substrates en route to degradation. In order to examine the relationship between these pathways, we generated an additional ERAD substrate (CD8^L*M*^) containing defects in both the luminal domain and the TMD by combining the CD8^TMD*^ and CD8^LUM*^ mutations. CD8^L*M*^ colocalised extensively with the ER markers BAP31 and V5-tagged ERp57 (also known as PDIA3) but not the Golgi marker GM130 (also known as GOLGA2) (Fig. S4D), and did not acquire Golgi modifications, but remained exclusively as the unmodified precursor ‘u’ (Fig. S4C). Thus, unlike CD8^TMD*^ and CD8^LUM*^, CD8^L*M*^ appears to be stringently retained in the ER. This indicates that distinct mechanisms recognise the defects in CD8^TMD*^ and CD8^LUM*^, and together have an additive effect resulting in stringent ER retention. CD8^L*M*^ was rapidly degraded during cycloheximide chase assays (Fig. 5E), was subjected to ubiquitylation (Fig. S4G) and was stabilised by proteasome inhibitor treatment (Fig. S4H,I), confirming that CD8^L*M*^ is an ERAD substrate. We then tested whether the presence of the misfolded luminal domain in CD8^L*M*^ could overcome the requirement for ubiquitylation of the cytoplasmic region during ERAD. However, as seen for CD8^TMD*^, replacing the cytoplasmic lysine residues with arginine residues inhibited ubiquitylation (Fig. S4G, compare lanes 4 and 8) and caused a dramatic stabilisation of CD8^L*M*^ (Fig. 5E,F). Thus, introduction of the non-native TMD into CD8^LUM*^ in fact directed the protein towards a different ERAD pathway that required ubiquitylation of cytoplasmic lysine residues for degradation. This is interesting because it indicates that, in the context of CD8, the presence of a non-native TMD constitutes a dominant signal that commits the protein to a specific degradation pathway distinct from that which mediates degradation of substrates with solely luminal folding defects.

**Fig. 6.**
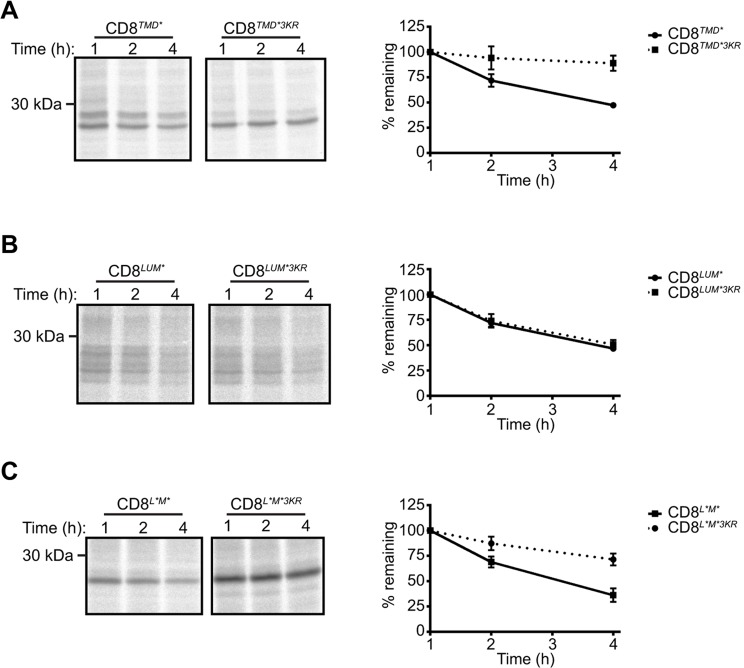
Cytoplasmic lysine residues are required for ERAD of CD8^TMD*^ and CD8^L*M*^ but not CD8^LUM^*. (A-C) Cells expressing the indicated chimeras were pulse-labelled with [^35^S]Met/Cys for 60 min and chased for up to 4 h in the presence of unlabelled Met and Cys. Radiolabelled CD8 was immunoprecipitated with anti-HA antibodies, and analysed by SDS-PAGE and phosphorimaging. Graphs show the amount of radiolabelled CD8 remaining at each time point expressed relative to that present after 1 h. Data represent the mean±s.e.m. of three independent experiments.

Although the conditions used for the cycloheximide chase assays did not induce ER stress or apoptosis (data not shown), treatment with cycloheximide might deplete short-lived ERAD factors. In order to rule out the possibility that the increased stability of CD8^TMD*3KR^ and CD8^L*M*3KR^ was due to depletion of factors required for degradation of these ERAD substrates (but not the other substrates examined), we examined the turnover of each of the substrates by radioactive pulse-chase assays (Fig. 6). Although the absolute rates of degradation measured using this approach were different to those obtained from cycloheximide chase assays, the requirement for lysine residues in the cytoplasmic tail was strikingly consistent. Hence, whereas mutation of lysine residues in the cytoplasmic tail markedly stabilised CD8^TMD*^ and CD8^L*M*^ (Fig. 6A,B), the mutant possessing only a luminal defect, CD8^LUM*^, continued to be rapidly degraded in the absence of cytoplasmic lysine residues (Fig. 6C).

**Fig. 7.**
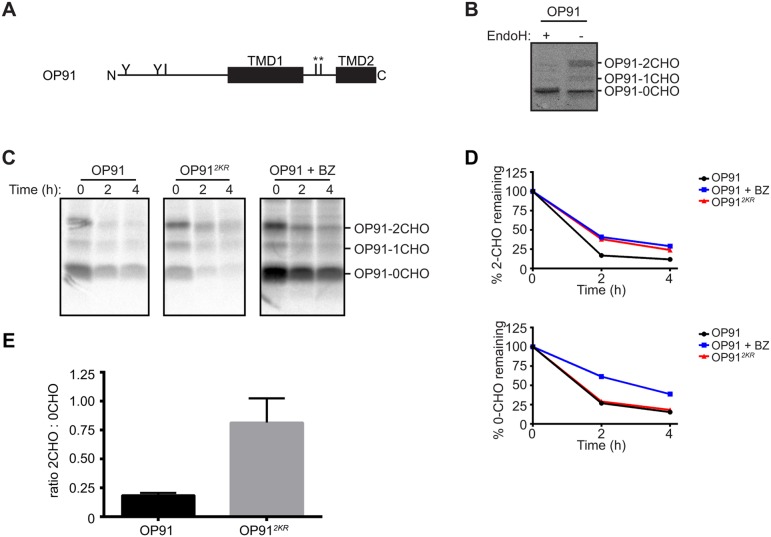
**Cytoplasmic lysine residues are required for degradation of OP91, an ERAD substrate containing an unassembled TMD.** (A) Schematic of the domain structure of OP91. Approximate location of N-glycosylation sites (Y) and lysine residues (I) are shown. * indicates cytoplasmic lysine residues replaced in OP91*^2KR^*. (B) Lysates of HeLa cells transiently expressing OP91 were treated with EndoH or mock treated, then analysed by SDS-PAGE and immunoblotting with anti-opsin antibodies. (C) Cells expressing OP91 or OP91*^2KR^* were pulse-labelled with [^35^S]Met/Cys for 60 min and chased for up to 4 h. OP91 was immunoprecipitated with anti-opsin antibodies and analysed by SDS-PAGE and phosphorimaging. Where indicated, cells were treated with bortezomib (BZ) throughout the pulse-chase labelling. (D) The amount of radiolabelled OP91 remaining at each time point was expressed relative to that present at the start of the chase. The glycosylated (2CHO) and non-glycosylated (0CHO) forms were quantified separately. Data represents the mean of two independent experiments. (E) The ratio of double glycosylated (2CHO) relative to non-glycosylated (0CHO) OP91 and OP91*^2KR^* at steady state was quantified by immunoblotting with anti-opsin. The graph represents the mean±s.e.m. of four independent experiments. (C) The ratios of the double glycosylated ‘-2CHO’ to non-glycosylated ‘-0CHO’ forms of OP91 and OP912KR were quantified at t=0 (steady state). The graph represents the mean±s.e.m. of four independent experiments.

In order to test whether these findings can be extended to other proteins containing non-native TMDs, we utilised OP91, a truncated form of the GPCR rhodopsin composed of the first and part of the second TMD (Fig. 7A). A proportion of OP91 is integrated into the ER membrane and undergoes N-glycosylation (Wunderley et al., 2014; Fig. 7B), and is subsequently degraded through a proteasomal pathway, suggesting it is a substrate for ERAD (Fig. 7C,D). As was seen for CD8^TMD*^, replacing the two cytoplasmic lysine residues in OP91 with arginine residues caused a marked stabilisation of the resulting protein OP91*^2KR^* (Fig. 7C,D). The lysine to arginine mutation specifically stabilised the N-glycosylated (and thus ER-integrated) forms of OP91 (Fig. 7C, OP91-1CHO and OP91-2CHO; Fig. 7D) but not the non-glycosylated form of the protein (Fig. 7C, OP91-0CHO; Fig. 7D). This is an important observation as it shows that the two cytoplasmic lysine residues are specifically required for ERAD of membrane-integrated OP91, but are not essential for proteasomal degradation of this polypeptide per se. These results provide further support for our hypothesis that ERAD substrates containing TMD defects are degraded through a distinct pathway that depends upon ubiquitylation of cytoplasmic lysine residues.

## DISCUSSION

**Fig. 8.**
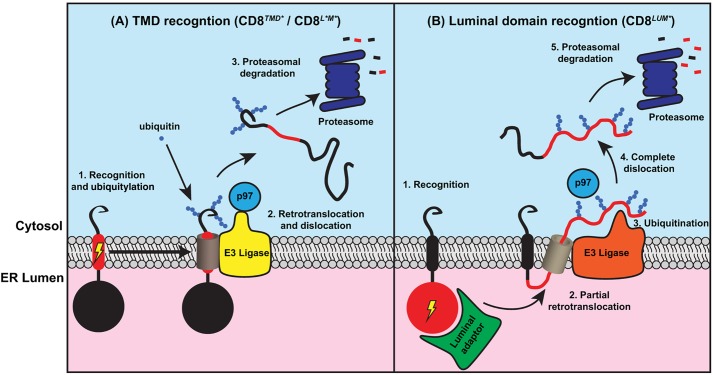
**Proposed model for degradation of CD8 chimeras containing defective transmembrane and/or luminal domains.** (A) Recognition of proteins containing aberrant TMDs mediates targeting to ERAD ubiquitylation and retrotranslocation complexes, possibly through a lateral gating mechanism. Ubiquitylation of the cytoplasmic domain of the ERAD substrate is then required for retrotranslocation and extraction from the membrane and targeting for degradation. (B) In contrast, proteins containing solely misfolded luminal domains are recognised by luminal ERQC factors and partially retrotranslocated, resulting in ubiquitylation of luminal domains and extraction into the cytoplasm for degradation.

The mechanisms by which proteins that contain defective TMDs are recognised and removed from the ER are poorly understood. Here, we generated a model protein to study TMD quality control by replacing the endogenous TMD of the type I membrane protein CD8α with an exogenous sequence derived from a polytopic membrane protein. The non-native TMD caused recognition by ERQC systems, leading to rapid degradation through the ERAD pathway. Degradation of CD8^TMD*^, as well as a second transmembrane ERAD substrate containing an unassembled TMD, was dependent upon the ubiquitylation of lysine residues within the cytoplasmic domain. In contrast, a version of CD8 containing the native TMD but a misfolded luminal domain (CD8^LUM*^) was efficiently degraded in the absence of cytoplasmic lysine residues. Our findings suggest that proteins with defective TMDs are removed from the ER of mammalian cells through a distinct ERAD pathway in which ubiquitylation of cytoplasmic residues is crucial for extraction from the ER membrane (Fig. 8).

In order for CD8^TMD*^ to represent a suitable model for studying the quality control of TMDs, it is important that the extracellular and luminal domains are folded, and several lines of evidence support this. Previous studies have shown that the luminal domain of CD8 folds independently of the rest of the molecule, and that replacing the TMD with exogenous sequences does not necessarily prevent transport to the cell surface (Li et al., 2010; Munro, 1995). In addition, the lack of recognition by BiP binding, recognition of the extracellular domain by anti-CD8 antibodies and the formation of interchain disulphide bonds all suggest that the luminal domain of CD8^TMD*^ was folded. Thus, we conclude that the major structural defect in this protein lies in the non-native TMD sequence.

The non-native TMD caused CD8^TMD*^ to be localised to the ER and targeted for degradation through the ubiquitin-proteasome system. This sequence, derived from the fourth TMD of PLP, contains five weakly polar residues and one highly polar residue that could potentially act as signals for ER localisation and ERAD (Houck and Cyr, 2011; Ng et al., 2012). In addition, residues located between the transmembrane and cytosolic or luminal domains might influence the behaviour of integral membrane proteins, and thus defects at the TMD junctions could also contribute to the recognition of CD8^TMD*^ by the ERQC machinery. Charged and polar residues within TMDs have long been thought to cause ER retention and degradation of membrane proteins, including unassembled T-cell receptor (TCR) subunits, the IgE receptor, membrane-bound IgM and several engineered proteins (Bonifacino et al., 1991, 1990; Cauvi et al., 2006; Fayadat and Kopito, 2003; Li et al., 2010; Williams et al., 1990). However, recent work suggests that in at least some cases, ER retention is due to translocation of the TMD into the ER lumen, leading to recognition by BiP and targeting for ERAD (Fayadat and Kopito, 2003; Feige and Hendershot, 2013; Shin et al., 1993). Our findings with CD8^TMD*^, which we show is stably integrated into the ER membrane, demonstrate that determinants embedded within the lipid bilayer can also lead to ER retention and ERAD of proteins containing non-native TMDs. Potential candidates for mediating TMD-based retention of CD8^TMD*^ include Rer1, calnexin and the E3 ligase Hrd1 (also known as SYVN1), which have been implicated in the ER retrieval, ER retention and ubiquitylation, respectively, of proteins containing non-native or misassembled TMDs (Cannon and Cresswell, 2001; Kaether et al., 2007; Li et al., 2010; Sato et al., 2009, 2003; Swanton et al., 2003). Future studies aimed at defining the role of these and other factors in ER retention and ERAD targeting of CD8^TMD*^ will provide new insight into the molecular basis for quality control of TMDs within the lipid bilayer.

In *S. cerevisiae*, proteins with misfolded membrane segments are degraded through a distinct ERAD-M pathway, which requires the E3 ligase Hrd1p, but not luminal factors, such as Yos9p, that target proteins with misfolded domains in the ER lumen for ERAD-L (Carvalho et al., 2006; Sato et al., 2009). It is not clear whether a similar distinction between ERAD pathways for proteins with transmembrane or luminal defects can be made in mammalian cells. Analysis of CD8^TMD*^ and CD8^LUM*^ allowed us to compare degradation of a single integral membrane protein containing defects in different regions of the polypeptide. We found that degradation of CD8^TMD*^ but not CD8^LUM*^ was dependent upon the presence of lysine residues in the cytoplasmic tail, suggesting that the location of a folding defect can influence the ERAD mechanism used. Replacement of cytoplasmic lysine residues with arginine residues inhibited ubiquitylation and extraction of CD8^TMD*^ from the ER membrane, consistent with the view that membrane-spanning ERAD substrates are initially ubiquitylated on domains located in the cytoplasm, leading to recruitment of p97 (also known as VCP), which pulls other regions of the protein across the ER membrane to the cytoplasm for degradation (Christianson and Ye, 2014; Ye et al., 2001). In contrast, degradation of CD8^LUM*^, which contains the native TMD but a misfolded luminal domain, was not dependent on cytoplasmic lysine residues, indicating that the site of substrate ubiquitylation might be determined by the position of the non-native domain, at least in the context of CD8 (see model in Fig. 8). CD8^LUM*^ might be ubiquitylated initially on lysine residues in the extracellular or luminal domain as recently shown for the unassembled MHC I heavy chain (Burr et al., 2013), or could potentially undergo non-canonical ubiquitylation of serine or cysteine residues in its cytoplasmic tail (Shimizu et al., 2010).

To our knowledge, the target sites for ubiquitylation have only been identified for three transmembrane proteins that are degraded by the cellular ERAD machinery. Nonetheless, these examples are consistent with a model whereby the location of the folding defect determines the initial site of ubiquitylation during ERAD. As observed for CD8^LUM*^, degradation of unassembled MHC I heavy chain, another type I membrane protein, does not require ubiquitylation of lysine residues in its cytoplasmic tail (Burr et al., 2013). Instead residues in its luminal domain are preferentially ubiquitylated during ERAD. The determinants for ERAD targeting were shown to lie solely within the luminal domain of the MHC I heavy chain, and it has been suggested that recognition by luminal adaptors initiates an early retrotranslocation event that exposes the luminal domain to the cytoplasm for ubiquitylation (Burr et al., 2013). In contrast, the type I membrane protein TCRα, which is targeted for ERAD owing to the presence of charged residues within the TMD, is ubiquitylated on residues in its cytoplasmic domain (Ishikura et al., 2010). If the TMD is translocated fully into the ER lumen, as recently suggested (Feige and Hendershot, 2013; Shin et al., 1993), both the structural defect and site for ubiquitylation would be located within the lumen. Sterol-induced ERAD of the polytopic membrane protein HMG-CoA reductase is dependent upon ubiquitylation of two lysine residues located in its cytoplasmic domains (Sever et al., 2003). ERAD targeting is dependent upon a series of sterol-regulated interactions between the TMDs of the regulatory protein Insig-1, HMG-CoA reductase and the ERAD E3 ligase gp78 (also known as AMFR), suggesting that the determinants for ERAD lie at least partly within the lipid bilayer (Lee et al., 2007; Sever et al., 2003). As observed for CD8^TMD*^, mutation of these specific lysine residues effectively blocks dislocation and degradation of HMG-CoA reductase (Sever et al., 2003). Finally, as we show here, degradation of OP91, an ERAD substrate containing an unassembled TMD, is also dependent upon cytoplasmically located lysine residues. These ERAD substrates therefore appear to fall into two distinct classes, those that possess TMD-based ERAD signals (CD8^TMD*^, OP91, HMG-CoA reductase) and require ubiquitylation of cytoplasmic residues, and those that contain luminal defects and do not (CD8^LUM*^ and MHC I heavy chain). The variable requirement for ubiquitylation of cytoplasmic lysine residues indicates that that these ERAD substrates are degraded by distinct ERAD mechanisms, either through distinct E3 ligases or alternatively by the same E3 ligase associated with different ERAD factors (Christianson et al., 2012).

On the basis of these observations, we propose a model whereby integral membrane proteins containing non-native determinants within their TMD(s) are recruited to ERAD complexes that mediate ubiquitylation on cytoplasmic regions, providing a handle for p97-mediated extraction and proteasomal degradation (Fig. 8A). In contrast, membrane proteins with folding defects in their luminal domain(s) might be targeted to ERAD complexes that mediate initial retrotranslocation of a luminal region of the polypeptide prior to its ubiquitylation and recruitment of p97 (Fig. 8B). The latter mechanism is conceptually similar to that which operates for soluble ERAD substrates (Christianson and Ye, 2014), and, as shown for unassembled MHC I heavy chain (Burr et al., 2013), is likely to involve recognition of the misfolded luminal domain by luminal adaptors such as OS-9 and XTP3-B. How proteins with TMD defects are targeted for ubiquitylation is not known, but this process could potentially involve direct recognition of signals within the bilayer by membrane-spanning E3 ligase complexes as has been shown for the Hrd1p in *S. cerevisiae* (Sato et al., 2009).

Interestingly, we found that ERAD of CD8-containing defects in the TMD and in the luminal domain (CD8^L*M*^) required ubiquitylation of cytoplasmic lysine residues. Hence, the TMD-located ERAD signal appears to be dominant in the context of this type I membrane protein. During membrane protein biosynthesis, the folding of domains within the cytoplasm, membrane and ER lumen might be interdependent (Skach, 2009), and therefore it is likely that some ERAD substrates will have defects located in more than one region of the polypeptide. Future work will be aimed at identifying whether the proposed model can be extended to explain ERAD of other misfolded membrane proteins in mammalian cells, and defining the molecular mechanisms and components that mediate degradation of proteins with defective TMDs.

## MATERIALS AND METHODS

### Reagents and antibodies

Antibodies against CD8 and rabbit HA were from Sigma, antibodies against BAP31, LAMP1, actin, Hsp70 and β-tubulin were from AbCam, anti-ERGIC53 antibody was from Alexis, mouse anti-HA antibody was from Santa Cruz Biotechnology, anti-BiP antibody was from Cell Signaling, anti-CNX and -CRT antibodies for immunoblotting were from Stressgen, anti-CRT antibody for immunofluorescence was from Thermo Scientific and anti-GM130 antibody was from BD Biosciences. Antibodies against opsin and STT3B antibodies were provided by Stephen High (University of Manchester, Manchester, UK). IRDye 800 CW and IRDye 680 RD were from LI-COR, and secondary antibodies for microscopy were from Jackson Laboratories (Stratech Scientific). The inhibitors leupeptin (Enzo Life Sciences), pepstatin A (Sigma), Z-LLF-CHO (PSII, Calbiochem), chloroquine (Sigma) and cycloheximide (CHX, Sigma) were used at 0.5 mM, 1 µg/ml, 10 µM, 5 mM and 100 µg/ml, respectively.

### DNA constructs

CD8^TMD*^ was generated by PCR overlap extension using human CD8α and human PLP as templates to insert the TMD sequence LFIAAFVGAAATLVSLLTFMIAATYNFAVL, and was cloned into pcDNA5/FRT/TO (Invitrogen). OP91, an N-terminal fragment (residues 1–91) of bovine rhodopsin (Wunderley et al., 2014), was provided by Stephen High. Other constructs were generated by site-directed mutagenesis, and were verified by DNA sequencing.

### Cell culture, transfection and stable cell line generation

To generate stable cell lines, HeLa TRex Flp-In host cells (provided by Stephen Taylor, University of Manchester, Manchester, UK) were transfected with CD8 variants. Stably transfected cells were selected using hygromycin B (ForMedium) and blasticidin (InvivoGen). Cell lines were maintained in Dulbecco's modified Eagle's medium (DMEM; Sigma) supplemented with 10% fetal bovine serum (FBS), 2 mM L-glutamine and 1% non-essential amino acids at 37°C and under 8% CO_2_. Experiments were performed after inducing expression with 1 µg/ml tetracycline for 16–20 h unless otherwise stated. For transient transfections, HeLa cells were transfected using Lipofectamine LTX (Invitrogen) and analysed after 16–20 h.

### Cycloheximide chase analysis of protein stability

Cells were treated with 100 µg/ml cycloheximide (CHX) to inhibit protein synthesis, and harvested immediately or at 60-min intervals following addition of CHX, by lysing directly in SDS-PAGE sample buffer (30 mM Tris-HCl pH 7.6, 2% SDS, 5% glycerol, 0.01% Bromophenol Blue and 100 mM DTT). Where indicated, inhibitors were added at 2 h prior to CHX and included throughout the chase. Samples were analysed by immunoblotting with anti-HA and anti-actin or -α-tubulin antibodies followed by IRDye-conjugated secondary antibodies and visualised using an Odyssey^®^ Sa Infrared Imaging System (LI-COR). Anti-HA antibody signal intensity was quantified and normalised relative to the loading control then expressed as a percentage of that present at the start of the chase. For EndoH treatment, cells were lysed in sample buffer and incubated with EndoH (1000 U/ml) (New England Biolabs) at 37°C overnight.

### Radiolabelling and pulse-chase analysis

Cells were grown in DMEM lacking Met and Cys (GIBCO) for 30 min, and then pulse-labelled in DMEM containing 22 µCi/ml [^35^S]Met/Cys EasyTag™ EXPRESS35S protein labelling mix (PerkinElmer) at 37°C for 5–10 min for protein maturation or 60 min for protein degradation assays, then chased in complete DMEM supplemented with 10 mM unlabelled Met and Cys for up to 90 min at the indicated temperature for protein maturation or up to 4 h at 37°C for protein maturation. At each time point, cells were lysed in IP-Tx buffer [10 mM Tris-HCl pH 7.6, 140 mM NaCl, 1 mM EDTA, 1% (v/v) Triton X-100 and 1 mM PMSF]. Lysates were clarified by centrifugation at 15,000 ***g*** for 10 min at 4°C, and immunoprecipitated with anti-HA antibodies and protein-A–Sepharose (Genscript). Immunoprecipitated material was analysed by SDS-PAGE and phosphorimaging.

### Detection of substrate ubiquitylation

Cells were induced with tetracycline for 16 h, and treated with or without PSII (10 µM) for 8 h. Cells were incubated in PBS containing 20 mM NEM for 5 min then lysed in Ub-IP buffer [25 mM Tris-HCl pH 7.4, 150 mM NaCl, 0.5% (w/v) Na-deoxycholate, 1% (v/v) Triton X-100 (2% for CD8^LUM*^), 0.1% (w/v) SDS, 5 mM NEM and 1 mM PMSF], and lysates were incubated on ice for 1 h with intermittent vortexing. Lysates were clarified by centrifugation at 5000 ***g*** for 10 min at 4°C, then immunoprecipitated with anti-HA antibody followed by protein A or G sepharose beads. Immunoprecipitated material was analysed by SDS-PAGE (10% polyacrylamide gels) and immunoblotting with anti-ubiquitin antibody.

### Carbonate extraction

Cells were trypsinised and resuspended in HIM buffer (10 mM HEPES pH 7.5, 200 mM mannitol, 70 mM sucrose, 1 mM EGTA and 1 mM PMSF), then homogenised by being passed 20 times through a 25G needle. Extracts were centrifuged at 1500 ***g*** for 15 min, and the supernatant spun at 100,000 ***g*** for 30 min. Membrane pellets were subjected to two rounds of carbonate extraction, consisting of 1 h incubation on ice in 200 µl of 100 mM NaCO_3_ followed by spinning at 100,000 ***g*** for 1 h. The final membrane pellet and each supernatant were analysed by SDS-PAGE.

### Immunofluorescence microscopy

Cells were fixed for 15 min in 3% formaldehyde (Sigma), quenched with glycine and permeabilised for 4 min in 0.1% (v/v) Triton X-100 in PBS. Cells were labelled with primary antibodies for 30–60 min followed by Alexa-Fluor-594- or Alexa-Fluor-488-conjugated secondary antibodies for 30 min. Coverslips were mounted in ProLong Gold with DAPI (Molecular Probes) and viewed with an Olympus BX60 upright microscope using a 60×1.40 N.A. PlanApo objective. Images were taken with a CoolSNAP EZ camera (Photometrics) using MetaMorph software (MDS Analytical Technologies). All image processing was performed using ImageJ (http://rsbweb.nih.gov/ij/).

### MTT assay of viable cells

Cells were seeded at 8000 cells/well in a 24-well dish, and induced with tetracycline at 24-h intervals for a maximum of 96 h. Cells were incubated for 2 h in serum-free DMEM containing 0.5 mg/ml MTT, and the resulting formazan crystals dissolved in 500 µl DMSO. Triplicate samples were transferred into a 96-well plate and the absorbance at 570 nm measured using a Synergy H1 Hybrid reader (BioTek).
